# Evidence of the two surface states of (Bi_0.53_Sb_0.47_)_2_Te_3_ films grown by van der Waals epitaxy

**DOI:** 10.1038/srep03406

**Published:** 2013-12-03

**Authors:** Liang He, Xufeng Kou, Murong Lang, Eun Sang Choi, Ying Jiang, Tianxiao Nie, Wanjun Jiang, Yabin Fan, Yong Wang, Faxian Xiu, Kang L. Wang

**Affiliations:** 1 Department of Electrical Engineering, University of California, Los Angeles, California 90095, USA; 2 National High Magnetic Field Laboratory, Tallahassee, FL 32310, USA; 3 Center for Electron Microscopy and State Key Laboratory of Silicon Materials, Department of Materials Science and Engineering, Zhejiang University, Hangzhou, 310027, China; 4 State Key Laboratory of Surface Physics and Department of Physics, Fudan University, Shanghai 200433, China; 5 These authors contributed equally to this work.

## Abstract

The discovery of topological insulators (TIs) has led to numerous exciting opportunities for studying topological states of quantum physics and for exploring spintronic applications due to the new physics arising from their robust metallic surface states. Here, we report the high-quality topological insulator (Bi_x_Sb_1−x_)_2_Te_3_ thin films using a single van der Waals GaSe buffer layer. As a result, ultra-low surface carrier density of 1.3 × 10^12^ cm^−2^ and a high Hall mobility of 3100 cm^2^/Vs have been achieved for (Bi_0.53_Sb_0.47_)_2_Te_3_. The high-quality films enable us to observe quantum oscillations associated with the top and bottom surface states and to manipulate the Dirac electrons and bulk holes' conduction properties. The observation of the two surface states may lead to a path towards the implementation of TIs in spintronics.

The discovery of two and three dimensional topological insulators (TIs) has generated strong activities in the condensed matter physics community^1^,^2^,^3^,^4^,^5^. Due to their Dirac-cone-like surface states and their relatively large bulk band gap, most research of 3D TIs has been based on Bi_2_Se_3_ and Bi_2_Te_3_^5^,^6^,^7^,^8^,^9^,^10^,^11^. Despite the progress made to date, limited success has been achieved in reducing defects which causes high bulk conduction, overwhelming the surface charge transport. Only very recently, the research on ternary compound TIs, such as BiTe_2_Se, BiSe_2_Te, (Bi_x_Sb_1−x_)_2_Te_3_ and (Bi_x_Sb_1−x_)_2_Se_3_^12^,^13^,^14^,^15^,^16^,^17^, has demonstrated enhanced and tunable Dirac surface over bulk conduction. Among them, ternary compound of (Bi_x_Sb_1−x_)_2_Te_3_ is particularly interesting, because it reaches a record low 2D carrier density of 2 × 10^11^ cm^−2^ in a 6 nm thin film^15^. And this compound with Cr-doping^18^ is also responsible for the newly demonstrated quantum anomalous Hall (QAH) effect.

Here, we report the use of a single GaSe van der Waals buffer layer on GaAs (111)B substrate to improve the growth of (Bi_x_Sb_1−x_)_2_Te_3_. The transport measurements show a clear ambipolar gating effect^14^ with top gate biases for all composition *x*, and a high Hall mobility of 3100 cm^2^/Vs from the surface electrons is observed. Shubnikov-de Haas (SdH) oscillations associated with the top and bottom surface states can also be observed and distinguished by the top gate biases. The results suggest that the van der Waals gap between the GaSe and (Bi_x_Sb_1−x_)_2_Te_3_ made it possible to observe the transport properties of the bottom Dirac Ferimions of (Bi_x_Sb_1−x_)_2_Te_3_, and to improve the overall film quality.

## Results

### Cross-sectional TEM and EDX of (Bi_0.53_Sb_0.47_)_2_Te_3_

**Figure 1:**
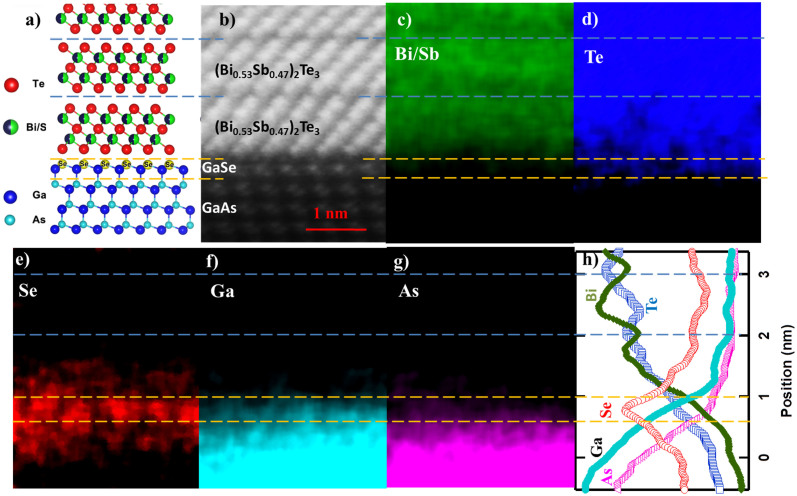
Cross-sectional TEM and EDX of (Bi_0.53_Sb_0.47_)_2_Te_3_. (a) A schematic diagram of the atomic layer structures of the TI film, interface and substrate. (b) High resolution TEM image exhibits QL of (Bi_0.53_Sb_0.47_)_2_Te_3_ film, GaAs substrate and the atomically sharp interface. Each QL is marked by the blue lines. A single GaSe layer is marked by the orange lines. (c–g) Distribution maps of individual elements: Bi/Sb (c), Te (d), Se (e), Ga (f) and As (g). (h) Average intensity profile of (b). The sharp peak of Se (red circles) confirms the presence of GaSe single layer.

For (Bi_x_Sb_1−x_)_2_Te_3_ illustrated in Figure 1a, we first show the cross sectional high resolution TEM in Figure 1b. The quintuple layers (QLs) of TI films are clearly revealed on top of the GaAs substrate. Within each QL, five atomic layers are observed. A slightly darker gap represents the van der Waals gaps between TI quintuple layers, marked by the dashed blue lines in Figure 1b. We also notice there is a similar gap between the TI film and the substrate, which is consistent with the van der Waals epitaxy growth mode^19^,^20^. At the interface, the top atomic layer shows slightly brighter dots, compared with the rest of GaAs substrate, implying the presence of other heavier atoms. This is probably due to the fact that the top layer of As were substituted by Se during the de-oxidization annealing under Se-rich environment; a more solid evidence will be given by the space resolved EDX in Figure 1c–h. Since GaSe is a quadruple layered structure, with Se-Ga-Ga-Se alternative atomic layers along the c-axis^21^, and the coupling between two quadruple layers is predominantly of the van der Waals type. After the formation of GaSe buffer layer, the top most Se atoms have no dangling bands, thus promoting the van der Waals epitaxial growth of TI films^22^.

To confirm the TI-GaSe-GaAs configuration (as shown in Figure 1a), energy dispersive X-ray spectroscopy (EDX) mapping in the Cs-corrected STEM (FEI TITAN) has been performed in this cross-section area. The distribution maps of each individual element are shown in Figure 1c–g. All the elements display a distinct distribution pattern: Bi, Sb and Te are located on the top half of the map; Se is located at the interface region; Ga and As are located in the bottom half. Specifically Figure 1h exhibits the average intensity profile for each element in Figure 1b; we can clearly see a sharp Se peak (red circles) at the film/substrate interface and Ga (blue dots) also extends to the interface area. Hence, this supports the former statement that a GaSe buffer is developed at the interface. We also notice that Bi (green triangles) and Te (blue squares) exhibit oscillating intensities with opposite phases. Bi demonstrates a peak in the center of TI layers (green line in Figure 1h), while Te shows a dip (blue line in Figure 1h), which could be understood as the following: for each QL of Te-Bi(Sb)-Te-Bi(Sb)-Te (Fig. 1b), Bi is accumulated in the center, while Te along the edges. The high crystal quality of the TI films also demonstrates exceptional electric properties as we will show below.

### Ambipolar conduction effect of (Bi_0.53_Sb_0.47_)_2_Te_3_

(Bi_x_Sb_1−x_)_2_Te_3_ is a non-stoichiometric alloy with Bi atoms randomly replaced by Sb, as illustrated in Figure 1a. We have studied the electronic properties of the thin films with various Bi concentration x, ranging from 0.32 to 0.77. Ambipolar effects have been observed for all the samples under gate biases (Supplementary Figure S2). At *V_G_* = 0 V, the samples demonstrate a transition from p-type to n-type as the Bi concentration increases. At x = 0.53, the lowest bulk carrier concentration is achieved with high carrier mobility.

For the 10 QL (Bi_0.53_Sb_0.47_)_2_Te_3_ sample, the longitudinal resistance *R_xx_* shows a semiconductor like temperature dependent relation with an activation energy of ~20 meV, estimated from the high temperature Arrhenius plot (supplementary Figures S4 inset), suggesting an impurity band positioned about 20 meV above the bulk valance band, similar to the reported value in bulk Bi_2_Te_2_Se^12^. *R_xx_* saturates to a constant value down to 0.3 K (supplementary Figure S3), implying a temperature independent surface conductance^10^. Similar R-T curves have been reported for other TI thin films or nano-plate^12^,^14^,^23^.

**Figure 2:**
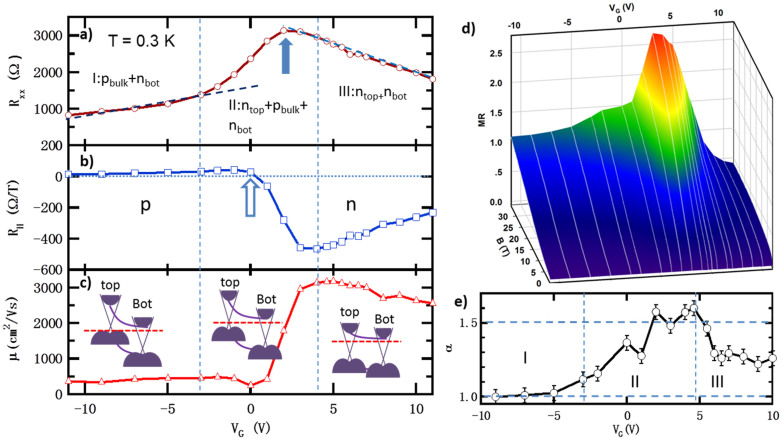
Gate dependent transport properties of (Bi_0.53_Sb_0.47_)_2_Te_3_ at 0.3 K. (a) Typical dependence of longitudinal resistance *R_xx_* on gate voltage *V_G_*. In regions I and III, the dependence is monotonically, while in region II, it shows a peak (solid arrow). (b) In regions I and III the monotonic change of *R_H_* as a function of *V_G_*, implies there exist a dominant carrier, holes or electrons, respectively. In region II, the non-monotonic behavior suggests a mixture of holes and electrons. (c) Gate dependent Hall mobility (*μ*), which exhibits a peak of 3100 cm^2^/Vs in the electron dominated region III. The insets show the schematic sketches of the band diagrams in the three regions. (d) Magnetoresistance, which also demonstrates a peak at *V_G_* = 3 V, coincident with *R_xx_* maximum. (e) Gate dependence of parameter α extracted from the fit to the HLN equation. Two plateaus of α = 1.0 and 1.5 in the regions, I and II, are evidenced.

At 0.3 K, typical gate voltage (*V_G_*) dependence of the longitudinal resistance (*R_xx_*) exhibits a broad peak (solid arrow in Figure 2a). It is about a few times greater than the resistance at large *V_G_* far from the peak position. The high field (6 T) Hall coefficients, *R_H_*, also reverse signs from negative to positive (hollow arrow in Figure 2b). This ambipolar field effect^14^,^24^ can be explained by a systematical change of the dominated conduction from the top and bottom surface electrons to bulk holes, which are confirmed from the Hall data as will be discussed later.

To explain transport data, we divide the range of top gate bias into three regions. In regions I (*V_G_* < −3 V) and III (*V_G_* > 4 V) (Figure 2a), bulk holes and surface electrons are the dominant carriers, respectively. The *R_xx_* shows a linear dependence of the gate voltage, as indicated by the dashed lines in Figures 2a. The electron density reaches the lowest value at *V_G_* = 4 V and furthermore, we also observe a high Hall mobility of 3100 cm^2^/Vs. In the mixed region II (−3 V < *V_G_* < 4 V), the total conductance crosses from top and bottom surface electrons (*n_top_* + *n_bot_*) to bulk holes and bottom surface electrons (*p_bulk_* + *n_bot_)*. As *V_G_* is decreased from region III, *n_top_* decreases and *R_xx_* increases accordingly; upon further decrease of *V_G_*, *p_bulk_* increases; hence the conductance of bulk holes increases, thus *R_xx_* reaches a peak. The change of conductance from electrons to holes is also evidenced as *R_H_* changes sign. This is accompanied by the dramatic decrease of the Hall mobility due to the much lower mobility of bulk holes. It is worth noting that the maximum in *R_xx_* does not completely coincide with that of the *R_H_* crossing zero^14^, nor with the *R_H_* maximum^25^. The small deviation may be attributed to the coexistence of three carriers conducting channels and the interplay of different carrier densities and their mobilities under different gate voltages.

The magnetoresistance (*MR* = *R_xx_*(*B*)/*R_xx_*(0) − 1) also exhibits a strong gate-voltage dependence. It has a single peak of 2.6 at *V_G_* = 3 V, coincident with the *R_xx_* maximum. A linear MR has also been observed in the bulk hole dominated region at high magnetic field, as indicated by dashed red lines in the Supplementary Figure S5, similar to other bulk dominated TI samples^11^,^26^,^27^. On the contrary, in the surface electron dominated region, linear MR is not observed.

At low magnetic field, sharp cusps of the weak antilocalization (WAL) effect can been seen. This weak antilocalization (WAL) effect is a signature of topological surface states associated with the helical states^28^,^29^,^30^,^31^,^32^,^33^. According to the Hikami-Larkin-Nagaoka (HLN) theory^34^, it can be described as 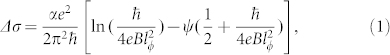
where Δ*σ* represents magneto conductivity, *l_ϕ_* is the phase coherence length, *Ψ* is the digamma function, and *α* equals ½ for a single coherent channel. For multiple independent parallel channels, *α* is equal to *n* × ½ and *n* is the number of conducting channels^27^. In our analysis, *α* shows a clear gate voltage dependence, as shown in Figure 2e.

A striking feature is that *α* is close to 1 in region I, suggesting that there coexist two separate conduction channels (bulk holes and bottom surface electrons). In the transition region II, *α* increases to 1.5 at around the *R_xx_* maximum, implying that bulk hole, top and bottom surface electrons constitute three separate channels. In region III, *α* decreases to around 1.3 suggesting the system has mostly electrons from top and bottom surfaces with some mixed bulk holes. We have noticed there are some discrepancies between our results and other people, who have an alpha value changes between 0.5 to 1 from p-type to n-type region, depending on gate voltages^35^,^36^. We believe this is because in their films, the Fermi level of the far side surface (top surface) is buried inside the bulk valence band, which will not contribute too much to the total conduction. On the contrary, in our films the Fermi level of the far side surface (bottom surface) is pined within the bulk band gap, thus it will contribute significantly to the conductance. Hence we have one more conducting channel compared with them. So *α* changes from 1 to 1.5 as gate voltage changes.

### Quantum oscillations from top and bottom surface states

**Figure 3:**
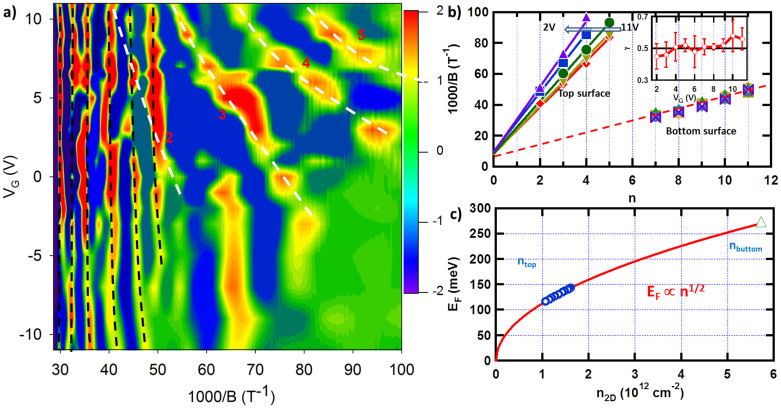
Shubinkov-de Haas oscillations from the top and bottom surface states. (a) *d*^2^*R_xx_*/*dB*^2^ as a function of 1/*B* and *V_G_*. Both gate dependent and independent peaks are observed. The features which change with *V_G_* originate from the formation of the Landau levels of Dirac fermions on the top surface states (white dashed lines, the Landau levels 2 to 5 are marked). The *V_G_*-independent features come from the Landau levels of the bottom surface states (black dashed lines). (b) Landau fan diagram of the peaks. The peaks of the top surface states (solid symbols) show systematic changes depending on the gate voltages, while that of the bottom surface states are almost constant. Inset: The intercept γ as a function of gate voltages. The black line indicates γ = 0.5. (c) The carrier density of the top (circles) and bottom (triangle) surface states as a function of Fermi energy *E_F_* extracted from the corresponding SdH oscillations for various gate voltages. A quadratic relationship is shown.

At 0.3 K, oscillations can be seen in the longitudinal resistance after substrate a smooth background, as shown in the supplementary figure S6, at various gate voltages. To emphases the gate dependent oscillations and to eliminate the MR background, we have used the second derivative of *R_xx_* (*d*^2^*R_xx_*/*dB*^2^) in Figure 3a. The first thing to notice is that there are gate voltage dependent peaks as accentuated by the white dashed lines. These peaks (valleys) originate from the formation of Landau levels of Dirac fermions on the top surface states, consistent with the data in Figure 2; the gate dependent shift is due to the decreasing of the top surface carrier density as the Fermi level approaches to the Dirac point. At the same time, there are other gate independent peaks at high magnetic field, as indicated by the black dashed lines. These peaks are attributed to those from the Landau levels of the bottom surface states. The strong screening effect arises from the high dielectric constant of TI materials prevents the bottom surface states from being affected by the gate bias^28^, resulting in little or almost no change of carrier density and correspondingly constant oscillation frequencies. It is also interesting to notice that at *V_G_* = 0 V, the top and bottom surface states have different oscillation frequencies (and carrier densities). This may be explained by the different band bending at the interface^28^, as indicated in the inset of Figure 2c. These two independent quantum oscillation frequencies arising from the top and bottom surface states are very rare to be seen^28^,^37^, because it requires very high quality for both two surface states.

The Landau fan diagram for various gate voltage values is plotted in Figure 3b, in which the 1/B values corresponding to the maximum in Figure 3a is plotted as a function of Landau level index *n*^12^,^26^,^38^,^39^. The solid symbols represent the top surface states, demonstrating a systematic shift depending on gate biases. The open symbols represent the bottom surface states, which show no dependence on gate bias. It is well known that in the SdH oscillations, the Landau level index n is related to the cross section area of the Fermi surface (SF) by 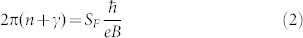
where *e* is the electron charge, 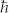 is the Plank's constant divided by 2*π*, *B* is the magnetic flux density, and *γ* = 1/2 or 0 represents the Berry phase of π or 0^40^. Linear fits yield intercepts at the abscissa of 0.51 ± 0.04, confirming the presence of massless Dirac Fermions carrying a π Berry phase. From the slopes, 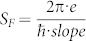, 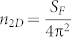, and 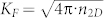 can be obtained for different gate bias voltages. Using the E-K diagram from ARPES results (supplementary Figure S8), the Fermi energy *E_F_* can also be estimated based on *K_F_*.

Figure 3c displays the surface carrier density *n* as a function of Fermi energy *E_F_*. The *n_top_* is effectively tuned from 1.61 × 10^12^ down to 1.07 × 10^12^ cm^−2^ by sweeping the gate voltage from 11 V to 2 V. A quadratic relationship of *E_F_* ∝ *n*^1/2^ can be observed, as indicated by the red line, confirming the linear E-K relationship of the Dirac-cone.

## Discussion

In summary, to achieve high quality TI thin films, passivating the top surface of substrates^41^,^42^,^43^ is a crucial step. In our practice, we have passivated the GaAs (111)B substrates through the formation of a single GaSe buffer layer. On top of this, we demonstrated the van der Waals epitaxy growth of very high quality (Bi_0.53_Sb_0.47_)_2_Te_3_ thin films with atomically abrupt interface and without any interfacial layers. Superior transport properties with an ultra-low surface carrier density of 1.3 × 10^12^ cm^−2^ and a high Hall mobility of 3100 cm^2^/Vs were achieved. Because of the presence of this pristine bottom surface, quantum oscillations have been observed not only from the top, but also from the bottom surface states, evidenced by two different oscillation frequencies and different top gate bias dependence.

The improvement of material quality through the van der Waals epitaxy growth and especially the observation of the bottom surface states offer many exciting opportunities for new TI based experiments or devices. By adding additional bottom bias, top and bottom surface states can be separately tuned, and they can have opposite types of Dirac fermions. This can be used for studying the topological excitons condensation^44^,^45^, as well as the electric field controlled spin generator for spintronic applications based on the spin-momentum locking in the topological surfaces^45^.

## Methods

### Growth of (Bi_0.53_Sb_0.47_)_2_Te_3_ thin films

The GaAs (111)B substrates have been cleaned by acetone with ultrasonic for 10 minutes before loaded into the growth chamber. Then the substrates were annealed to 580°C and cooled to growth temperature, under Se rich environment. During this anneal procedure a strained GaSe single buffer layer was formed on the surface. The film growth was performed at about 200°C, with Bi, Sb, and Te shutters opened at the same time. The flux ratio of Bi:Sb:Te was about 1:4:20 for the compound of (Bi_0.53_Sb_0.47_)_2_Te_3_, ex-situ estimated by EDX. The growth mode was via layer-by-layer, monitored by the RHEED intensity oscillations (supplementary Figure S1), through which accurate film thickness could be achieved. There was a deposition of 2 nm Al immediately after the growth of TI film in MBE chamber to protect the film for environmental doping^32^.

### Device fabrication

The MBE-grown TI thin films were first patterned into a micron-scale Hall bar geometry using conventional optical photolithography and a subsequent CHF_3_ dry-etching of 15 s. Hall bar contacts were defined by photolithography and followed by *e*-beam evaporation of 10 nm Titanium (Ti) and 100 nm Gold (Au). A 15 nm-thick Al_2_O_3_ dielectric layer was conformally deposited by ALD at 250°C to serve as the high-k gate dielectric. Another step of photolithography was needed to open window, and dry etching was carried out to etch the Al_2_O_3_ in the contact area with subsequent dip in 5% diluted HF. Finally, the top-gate electrode and Hall channel contacts were defined and followed by metal deposition of Ti/Au (10 nm/100 nm).

### Electrical measurements

The devices were cooled down using a He3 insert at Cell 8 and 12 at the National High Magnetic Field Laboratory at Florida. The DC magnetic field used was up to 35 T and the base temperature was 0.3 K. The electrical characteristics were measured using a lock-in technique (magnetotransport) with a constant AC current of 0.1 μA at 13 Hz plus a 5 μA DC bias current by Keithley 6221. The gate bias was provided by a Keithley 2401.

### TEM characterizations

The observations of the atomic structure and atomic EDX mapping at the interface between (Bi_0.53_Sb_0.47_)_2_Te_3_ and GaAs were conducted using a FEI TITAN Cs - corrected ChemiSTEM™, operating at 200 kV. This instrument incorporates the spherical aberration corrector and ChemiSTEM technology, and can achieve the resolution to 0.08 nm. The cross-sectional samples for TEM were prepared by a dual beam™ by gallium ion milling (Quanta 3D, FEG, FEI). All parameters were carefully optimized to avoid the gallium injection.

## Author Contributions

L.H. and K.W. conceived the idea and supervised the overall research. L.H. and X.K. synthesized the thin films. M.L., T.N. and W.J. fabricated the devices. L.H., X.K. and E.S.C. carried out low-temperature transport measurements. Y.J. and Y.W. performed the structural analysis. L.H., Y.F. and F.X. contributed to the analysis. L.H., Y.F., F.X. and K.W. wrote the paper with helps from all other co-authors. L.H., X.K., and M.L. contributed equally to this work.

## Supplementary Material

Supplementary Information Supplementary information
