# Supplementary material for: Evidence of the two surface states of (Bi0.53Sb0.47)2Te3 films grown by van der Waals epitaxy
Source: Sci Rep. 2013 Dec 3;3:3406. doi: 10.1038/srep03406 (PMC3847703; doi:10.1038/srep03406)
Supplement: Supplementary Information [file srep03406-s1.doc]

**Supplementary materials for**

**Evidence of the two surface states of (Bi_0.53_Sb_0.47_)_2_Te_3_ films grown by van der Waals epitaxy**

Liang He,^1^,**^✝^**^*^ Xufeng Kou,^1,*^ Murong Lang,^1,*^ Eun Sang Choi^2^, Ying Jiang,^3^ Tianxiao Nie,^1^ Wanjun Jiang^1^, Yabin Fan^1^, Yong Wang,^3^ Faxian Xiu,^4^ and Kang L. Wang^1^**^✝^**

*^1^Department of Electrical Engineering, University of California, Los Angeles, California 90095, USA ^2^National High Magnetic Field Laboratory, Tallahassee, FL 32310, USA ^3^Center for Electron MicroscopyandState Key Laboratory of Silicon Materials, Department of Materials Science and Engineering, Zhejiang University, Hangzhou, 310027, China ^4^State Key Laboratory of Surface Physics and Department of Physics, Fudan University, Shanghai 200433, China*

^*^ These authors contribute equally to this work.

**^✝^**To whom correspondence should be addressed. E-mail: [*wang@ee.ucla.edu*](mailto:wang@ee.ucla.edu)*, liang.*[*heliang@gmail.com*](mailto:heliang@gmail.com)

**Supplementary Information**

1. **Figure S1| MBE growth of (Bi_0.53_Sb_0.47_)_2_Te_3_**
2. **Figure S2| Gate dependent transport measurement of (Bi_x_Sb_1-x_)_2_Te_3_ for various composition x from 0.32 to 0.77**
3. **Figure S3| Gate dependent Hall resistance of (Bi_0.53_Sb_0.47_)_2_Te_3_**
4. **Figure S4| Resistance vs. Temperature curve of (Bi_0.53_Sb_0.47_)_2_Te_3_**
5. **Figure S5| Gate dependent MR of (Bi_0.53_Sb_0.47_)_2_Te_3_**
6. **Figure S6| Gate dependent *ΔR_xx_* vs. *1/B***
7. **Figure S7| *R_xx_*, *dR_xx_/dB* and *d^2^R_xx_/dB^2^* as function of** $B_{\perp}$
8. **Figure S8| ARPES image of (Bi_0.53_Sb_0.47_)_2_Te_3_**

**Figure S1**| **MBE growth of (Bi_x_Sb_1-x_)_2_Te_3_. (a)** RHEED image of GaAs (111)B substrate after annealed at 580°C for 5 min under Se rich environment. A Single layer of GaSe has formed on the surface. **(b)** RHEED image after 10 QL (Bi_0.53_Sb_0.47_)_2_Te_3_. The d-spacing between the two 1^st^ order streaky lines (indicated by the double arrows in **a** and **b**) has been used to estimate the film lattice constant. **(c)** The thickness evolution of lattice constant of a (Bi_0.53_Sb_0.47_)_2_Te_3_ film. The lattice relaxation happens within the first QL, consistent with the van der Waals growth mode. **(d)** RHEED intensity oscillations of the specular peak, from which growth rate of 163 s/QL has been estimated.

**Figure S2| Gate dependent transport measurements of (Bi_x_Sb_1-x_)_2_Te_3_ for various composition x from 0.32 to 0.77. (a)** Longitudinal resistance *R_xx_* versus gate voltage *V_G_*. All the samples, except x = 0.77 sample, exhibit ambipolar effect, suggesting the multiple conducting channels with different carrier type. At *V_G_* = 0 V, the samples demonstrate a transition from p-type to n-type as composition x changes from 0.32 to 0.77. This is consistent with the results of n-type Bi_2_Te_3_ and p-type Sb_2_Te_3_ in MBE grown thin films. **(b)** The Hall slopes of films, also shows a transition from p-type to n-type dependent on the gate voltage, suggesting a systematic shifting of Fermi level.

**Figure S3| Gate dependent Hall resistance of (Bi_0.53_Sb_0.47_)_2_Te_3_. (**a) Hall resistance *R_xy_* at various gate voltages *V_G_*. Clearly, the slopes change from negative to positive as the gate voltages decrease, representing the dominant carriers change from electrons to holes. The non-linearity of *R_xy_* suggests that more than one conducting channels exist in the system. (b) Zoom-in view demonstrates the linear dependence at low magnetic field. The Hall coefficient, *R_H_*, is calculated at B = 6 T.

**Figure S4| Resistance vs. Temperature curve of (Bi_0.53_Sb_0.47_)2Te_3_.** At low temperature ( *T* < 1.4 K) *R_xx_* is constant. Top inset: the Arrhenius plot of *Ln(R_xx_)* vs. *1000/T*. The estimated high temperature activation energy is 20 meV.

**Figure S5| Gate dependent MR of (Bi_0.53_Sb_0.47_)_2_Te_3_.** Magnetoresistance at various gate biases. **a**, As the gate decreases from 11 V to 3 V, the MR increases and reaches a maximum value at *V_G_* = 3 V, where the *R_xx_* also sits at the peak. **b**, MR decreases monotonically from 3 V to -11 V. A linear MR has also been observed at high magnetic field, as indicated by dashed red lines. It only happens in the region I, where the bulk holes are the majority carriers.

**Figure S6| Gate dependent *ΔR_xx_* vs. *1/B*.** Here, **w**e define *ΔR_xx_* = *R_xx_* - <*R_xx_*>, where <*R_xx_*> is a smoothed background. Quantum oscillations can be seen. To make the oscillations more clearly, in the manuscript, we have used the second derivative of *R_xx_*, *d^2^R_xx_/dB^2^*.

**Figure S7| *R_xx_*, *dR_xx_/dB* and *d^2^R_xx_/dB^2^* as function of $B_{\perp}$.** N corresponds to N-th Landau level that is crossing the Fermi energy *E_F_*, while n corresponds to N+1/2-th Landau level when *E_F_* is precisely between adjacent LLs.

It has been a little confusing some people use N and others use n to plot Landau fan diagram. But most recently, more people adopted n, in which they assign LL number n to the minimum of *R_xx_* [^1-3^](#_ENREF_1). In this manuscript, we assigned n to the maximum of *d^2^R_xx_/dB^2^*. This will give us the Onsager equation of . And for TI materials (π Berry phase), all the linear fitted curves should theoretically extrapolate to (0, ½).

**Figure S8| ARPES image of (Bi_0.53_Sb_0.47_)_2_Te_3_.** The electronic structure of the (Bi_0.53_Sb_0.47_)_2_Te_3_ thin film via ARPES. The Dirac cone can be clearly observed. The Fermi levels of top and bottom surface states extracted from SdH oscillations are shown in red and blue lines, respectively.

1. Qu, D.-X., Hor, Y.S., Xiong, J., Cava, R.J. & Ong, N.P. Quantum Oscillations and Hall Anomaly of Surface States in the Topological Insulator Bi2Te3. *Science* **329**, 821-824 (2010).

2. Analytis, J.G. et al. Two-dimensional surface state in the quantum limit of a topological insulator. *Nat Phys* **6**, 960-964 (2010).

3. Ren, Z., Taskin, A.A., Sasaki, S., Segawa, K. & Ando, Y. Large bulk resistivity and surface quantum oscillations in the topological insulator Bi_2_Te_2_Se. *Phys. Rev. B* **82**, 241306 (2010).
